# Pazopanib has equivalent anti-tumor effectiveness and lower Total costs than Sunitinib for treating metastatic or advanced renal cell carcinoma: a meta-analysis

**DOI:** 10.1186/s12885-019-5704-3

**Published:** 2019-05-23

**Authors:** Huan Deng, Yu Huang, Zhengdong Hong, Xuhui Yuan, Zhi Cao, Yiping Wei, Wenxiong Zhang

**Affiliations:** 1 Department of Thoracic Surgery, The First Affiliated Hospital of Nanchang University, Minde Rd, Nanchang, 330006 Jiangxi Province China; 2 Jiangxi Medical College, Nanchang University, Nanchang, 330006 China; 3 Department of Urology, The Second Affiliated Hospital of Nanchang University, Nanchang, 330006 China

**Keywords:** Meta-analysis, Pazopanib, Sunitinib, Targeted therapy, Renal cell carcinoma

## Abstract

**Background:**

Sunitinib and pazopanib are extensively used as first-line treatment of metastatic renal cell carcinoma (mRCC). We performed this meta-analysis to assess the anti-tumor effectiveness, toxicity, and total costs of the two drugs among patients with mRCC/advanced RCC (aRCC).

Materials and Methods: PubMed, ScienceDirect, Scopus, Web of Science, Ovid MEDLINE, the Cochrane Library, Embase, and Google Scholar were searched to obtain eligible articles. The endpoints included progression-free survival (PFS), overall survival (OS), adverse effects (AEs), and per-patient-per-month (PPPM) costs.

**Results:**

We included 14 medium- to high-quality studies. Both drugs were valid for mRCC/aRCC, with equivalent PFS (hazard ratio (HR) =1.06, 95% confidence interval [CI]: 0.98–1.15, *P* = 0.13), OS (HR = 0.92, 95% CI: 0.79–1.07, *P* = 0.29), objective response rate (ORR, risk ratio (RR) =1.03, 95% CI: 0.93–1.13, *p* = 0.58), and disease control rate (DCR, RR = 1.03, 95% CI: 0.94–1.22, *P* = 0.54). Sunitinib had more dosage reductions and higher PPPM (weighted mean difference = − 1.50 thousand US dollars, 95% CI: − 2.27 to − 0.72, *P* = 0.0002). Furthermore, more incidences of severe fatigue, thrombocytopenia, and neutropenia were recorded for sunitinib, but pazopanib had more liver toxicity. In subgroup analysis, studies from the US reported longer OS (HR = 0.86, 95% CI: 0.77–0.95, *P* = 0.004) and higher ORR (RR = 1.24, 95% CI: 1.03–1.51, *P* = 0.03).

**Conclusions:**

Pazopanib provides equivalent anti-tumor effectiveness and lower PPPM as compared with sunitinib for mRCC/aRCC. Great care should be given to pazopanib-treated patients with abnormal liver function. Nevertheless, more large-scale, high-quality studies are required.

**Electronic supplementary material:**

The online version of this article (10.1186/s12885-019-5704-3) contains supplementary material, which is available to authorized users.

## Background

Renal cell carcinoma (RCC) is the eighth most common type of tumor, with 65,340 cases and an expected 14,970 deaths in 2018 [1]. Moreover, more than 30% of patients have metastases when initially diagnosed [2]. The US Food and Drug Administration has approved sunitinib and pazopanib as first-line drugs for treating clear cell metastatic RCC (mRCC) [3, 4].

Sunitinib is an orally administered tyrosine kinase inhibitor (TKI) that has demonstrated efficacy and safety for mRCC in a randomized controlled trial (RCT) (NCT00130897) [5]. Pazopanib is also used as first-line TKI-targeted therapy for mRCC, and significantly promoted progression-free survival (PFS) and tumor response compared with placebo in patients with mRCC/advanced RCC (aRCC) in a randomized phase III clinical study [6]. Although both TKIs have shown superior benefits for treating mRCC, the best patient profile for the two drugs is still unclear. In a phase III RCT, Motzer et al. reported that pazopanib had comparable PFS and overall survival (OS) to sunitinib, but the safety outcomes favored the pazopanib group [7]. However, a phase II RCT demonstrated no significant difference in the total number of adverse events (AEs) between the sunitinib and pazopanib groups [8]. Other studies indicated that sunitinib was associated with better OS than pazopanib [9].

To address this dispute, we performed a meta-analysis of relevant articles to compare the anti-tumor effectiveness, AEs, and per-patient-per-month (PPPM) costs of pazopanib and sunitinib to provide evidence-based suggestions for patients with mRCC/aRCC in selecting first-line TKIs.

## Methods

We performed this meta-analysis in accordance with preferred reporting items for systematic review and meta-analysis (PRISMA) guidelines (Additional file 1: Table S1).

### Search strategy

PubMed, ScienceDirect, Scopus, Web of Science, Ovid MEDLINE, the Cochrane Library, Embase, and Google Scholar were searched up to September 2018 to select relevant articles comparing pazopanib versus sunitinib for mRCC/aRCC. The following terms were used: “pazopanib”, “sunitinib”, and “renal cell carcinoma”. The integral search in PubMed was: (pazopanib [MeSH Terms] OR pazopanib [Text Word] OR GW786034B [Text Word] OR Votrient [Text Word]) AND (sunitinib [MeSH Terms] OR sunitinib [Text Word] OR sutent [Text Word] OR SU011248 [Text Word]) AND (renal cell carcinoma [MeSH Terms] OR renal cell carcinoma [Text Word]). We also searched the references of included studies to identify further eligible articles. All included articles were written in English.

### Inclusion criteria

We included studies that met the following criteria: (1) patients were diagnosed with mRCC or aRCC (defined as regional lymph node metastasis and/or renal venous tumor thrombus and/or inferior vena cava tumor thrombus and/or adrenal metastasis or tumor infiltration with perirenal adipose tissue and/or renal sinus adipose tissue (but no more than the perirenal fascia), no distant metastasis of RCC); (2) pazopanib and sunitinib were compared; (3) results were PFS, OS, objective response rate (ORR), disease control rate (DCR), AEs, and PPPM; (4) written in English; (5) RCT or retrospective observational studies. We excluded reviews without raw data, meta-analysis, conference abstracts, case reports, and articles with repeated data.

### Data extraction

Two investigators (Deng and Zhang) extracted the following information independently: first author, country, year of publication, the number of patients in two groups, study design, patient characteristics (age, sex, study period, research subjects, pre-treatment), anti-tumor effectiveness indicators (PFS, OS, ORR, DCR), total number of grade 3–4 AEs and PPPM. The data about the total health care costs of pazopanib and sunitinib were converted into PPPM through mathematical operations that total health care costs was divided by the number of patients and the duration of treatment (month). A third researcher (Hong) settled disagreements under various circumstances. We used hazard ratios (HRs), which consider the number and time of events, instead of odds ratios to analyze PFS and OS. HRs and 95% confidence intervals (CIs) were obtained directly if Cox multivariate survival analysis was performed. Otherwise, HRs with 95% CIs were extracted from Kaplan–Meier curves in accordance with Tierney et al. [10].

### Quality assessment

We evaluated RCT quality using the 5-point Jadad scale, which includes questions on three major aspects: randomization, double-blinding, and withdrawals. A total score of ≥3 points indicated that a study was high-quality [11].

We evaluated the quality of retrospective observational studies using the 9-point Newcastle-Ottawa Scale, which includes a questionnaire on three major aspects: selection, comparability, and exposure. A total score of 8–9 points indicated that a study was high-quality; a total score of 6–7 points indicated that a study was medium-quality [12].

### Statistical analysis

We performed this meta-analysis using Review Manager (version 5.2) and STATA (version 12.0). HRs and 95% CIs were used to analyze PFS and OS (HR > 1 supports sunitinib group; HR < 1 supports pazopanib group). Risk ratios (RRs) and 95% CIs were used to analyze the ORR, DCR (RR > 1 supports pazopanib group; RR < 1 supports sunitinib group) and AEs (RR < 1 supports pazopanib group; RR > 1 supports sunitinib group). Weighted mean differences (WMD) with 95% CIs were used to analyze the PPPM. Subgroup analysis of PFS, OS, and ORR were performed to determine if these outcomes would vary according to country, the number of patients in pazopanib group, risk classification and study design. Heterogeneity was assessed using the χ^2^ test and *I*^*2*^ statistic. If *I*^*2*^ > 50% or *P* <  0.1, indicating significant heterogeneity, then the random-effects model would be used; if not, the fixed-effects model would be used. To enhance robustness, sensitivity analysis of PFS, OS, ORR, and DCR were performed to determine the effects of variables. We assessed publication bias through Begg’s test and Egger’s test. Additionally, if the bias caused by the combination of RCTs and retrospective studies was relatively small (for example, no significant difference was found in the subgroup analysis of PFS and OS about study design), we would analyze PFS and OS combining the two different types of studies. Otherwise, we would analyze RCTs and retrospective studies respectively. *P* <  0.05 was considered a significant statistical difference.

## Results

### Search results and quality evaluation

**Fig. 1:**
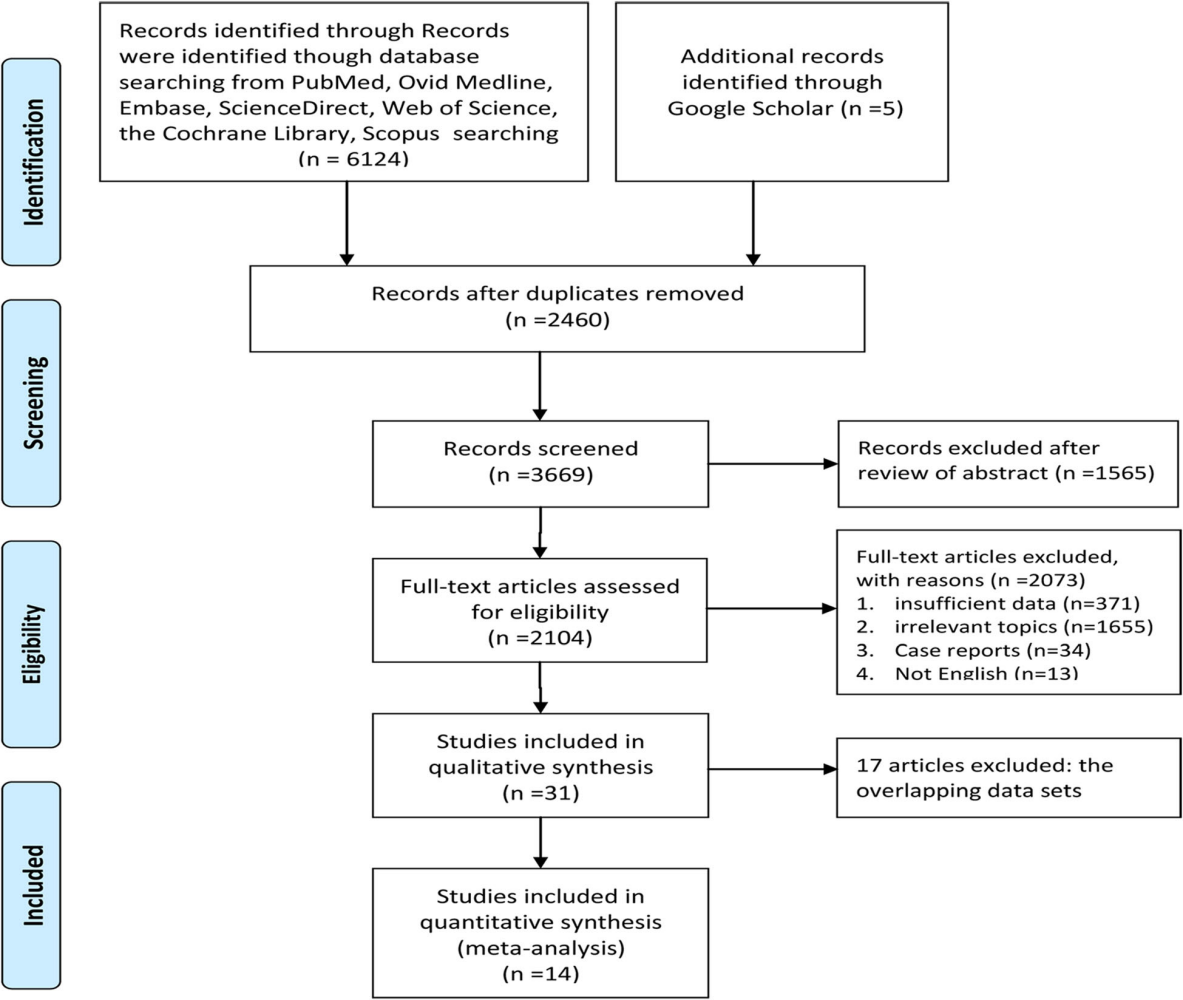
Flow chart of study selection

Figure 1 shows the study selection process. An eventual 14 studies involving 12,985 patients (pazopanib, 3047; sunitinib, 9938) were selected for this meta-analysis [7–9, 13–23]. Three studies were RCTs (two studies stemmed from the same RCT with different aspects of results: one reported anti-tumor efficacy and toxicity; the other reported economic data. They were considered as multiple reports representing one RCT) and 10 were retrospective observational studies. Eight articles were high-quality (three RCTs scored ≥3 points, one retrospective observational study scored 9 points, four retrospective observational studies scored 8 points). Five articles were medium-quality (four retrospective observational articles scored 7 points, one retrospective observational article scored 6 points). Table 1 lists the baseline characteristics and major evaluation indicators of all included articles.

**Table 1:** Characteristics of 14 included studies

Study	Publication year	Country	Study period	Research subjects	Study design	Data resource	Pazopanib No.	Sunitinib No.	Median age (y)	Pre-treatment	Median FU(m)	SQ
Motzer^b^ [7]	2013	USA	2008.8–2011.9	mRCC	RCT, phase III	14 countries in USA, Europe, Australia and Asia	557	553	61/62	NPT; RDT	20/20	4
Powles [8]	2012	UK	2008–2011	clear cell mRCC	RCT, phase II	Multi-institution in UK	34	43	67/73	No treatment	NA	3
Lalani [9]	2017	Canada	2011.1–2015.11	clear cell mRCC	RS	Canadian Kidney Cancer Information System database	93	577	65/64	NPT	NA	7
Kim [13]	2016	Korea	NA	mRCC with poor risk features	RS	Asan Medical Center	72	100	60/57	IMT; NPT	14.2/14.2	8
Byfield [14]	2015	USA	2009.10–2012.7	aRCC	RS	Optum Research Database and IMPACT National Benchmarking Database	84	84	63/61.5	RDT; NPT	6.0/6.0	7
Pal [15]	2017	USA	1993.1–2012.12.	aRCC	RS	The Surveillance, Epidemiology and End Results	89	545	68.9/68.1^a^	NPT	NA	8
Santoni [16]	2015	Italy	2005.1–2013.7	late-relapse clear-cell mRCC	RS	21 Italian centers	21	190	NA	RN	58.8/58.8	8
Vogelzang [17]	2017	USA	2006.1–2014.12	aRCC	RS	The 100% Medicare database and Part D	522	522	74/74.5	NA	15.7/15.7	9
Hansen^b^ [18]	2015	USA	2008.8–2011.9	mRCC	RCT, phase III	14 countries in USA, Europe, Australia and Asia	450	448	NA	NPT; RDT	20.0/20.0	4
Racsa [19]	2015	USA	2009.11–2012.1	aRCC	RS	claims data	90	193	67.9/68.2^a^	NA	12.0/12.0	7
Bianconi [20]	2016	Italy	NA	mRCC	RS	NA	19	78	68/64	renal surgery	NA	7
Escudier [21]	2014	France	NA	mRCC	RCT, phase IIIb	NA	86	82	64/62	NPT; RDT	NA	4
Ruiz-Morales [22]	2016	Canada	2005.1–2015.5	clear cell mRCC	RS	29 cancer centers in Canada, USA, Australia and so on	919	6519	65/62	NPT; IMT	NA	8
Kucharczyk [23]	2017	USA	2006.11.-2013.11	late-relapse clear cell mRCC	RS	A Single Institution in USA	11	4	NA	NPT	38.3/38.3	6

Abbreviations: mRCC: metastatic renal cell carcinoma, aRCC: advanced renal cell carcinoma, NPT: nephrectomy, RDT: radiotherapy, IMT: immunotherapy, RN: radical nephrectomy, RS: retrospective study, RCT: randomized controlled trial, NA: not available, SQ: score (RCT quality according to the Jadad scale and retrospective study quality according to the Newcastle-Ottawa scale)

^a^ Mean;

^b^ Two studies stemmed from one RCT with different aspects of results: one reported anti-tumor efficacy and toxicity; the other reported economic data

### Anti-tumor effectiveness

The anti-tumor effectiveness regarding PFS, OS, ORR, and DCR between the two groups was evaluated.

**Fig. 2:**
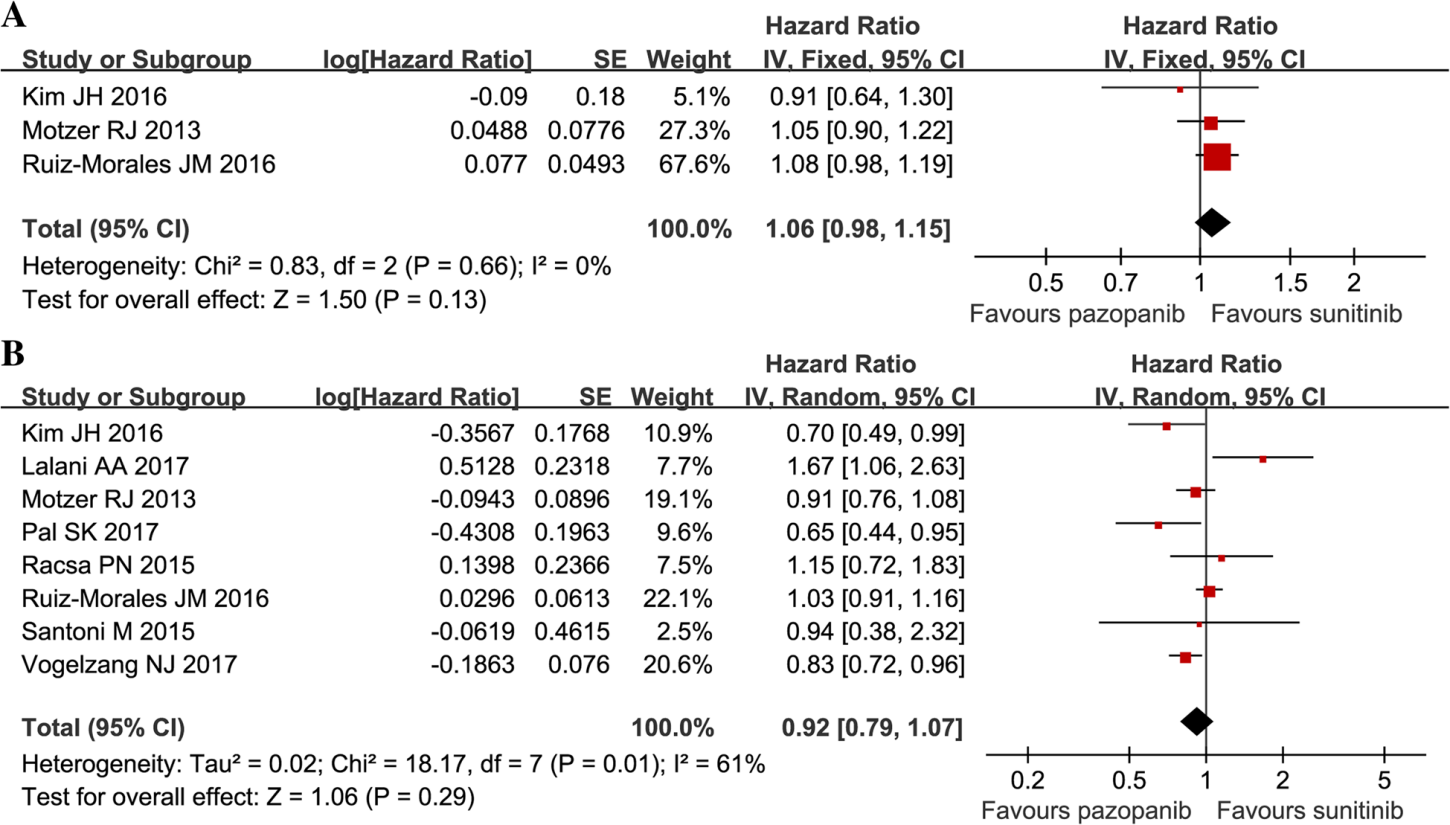
Forest plot of HR of PFS (**a**) and OS (**b**) associated with pazopanib versus sunitinib

Three articles compared the PFS (heterogeneity: *P =* 0.72, *I*^2^ = 0%). There was no significant difference between pazopanib and sunitinib (HR = 1.06, 95% CI: 0.98–1.15, *P =* 0.13; Fig. 2A).

Eight articles compared the OS (heterogeneity: *P* = 0.01, *I*^2^ = 61%). There was no significant difference between pazopanib and sunitinib (HR = 0.92, 95% CI: 0.79–1.07, *P* = 0.29; Fig. 2B).

**Fig. 3:**
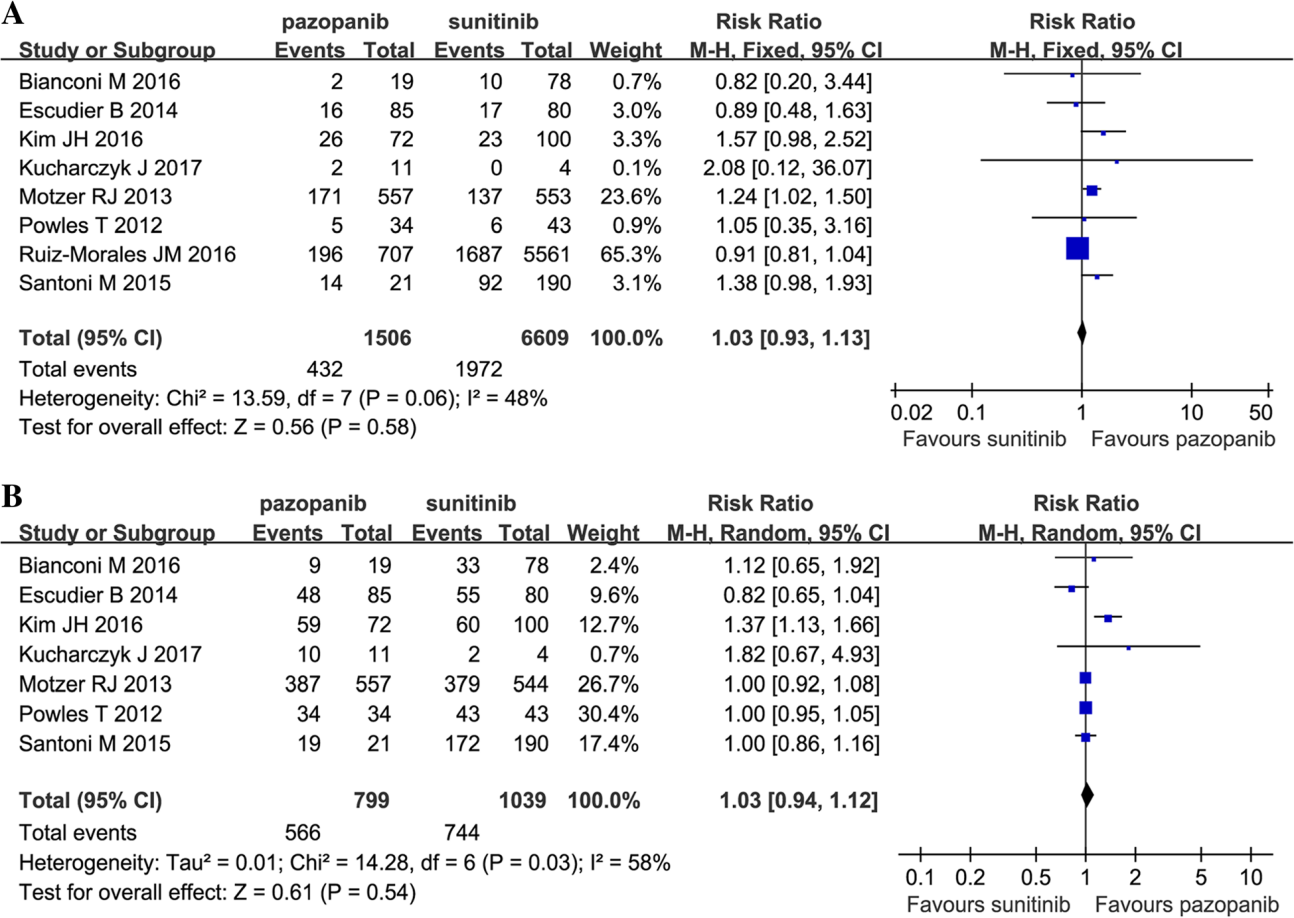
Forest plots of ORR (**a**) and DCR (**b**) associated with pazopanib versus sunitinib

Eight articles compared the ORR (heterogeneity: *P =* 0.06, *I*^2^ = 48%). There was no significant difference between pazopanib and sunitinib (RR = 1.03, 95% CI: 0.93–1.13, *P =* 0.58; Fig. 3A).

Seven articles compared the DCR (heterogeneity: *P =* 0.03, *I*^2^ = 58%). There was no significant difference between pazopanib and sunitinib (RR = 1.03, 95% CI: 0.94–1.22, *P =* 0.54; Fig. 3B).

### Toxicity

We compared grade 3–4 toxic events and performed subgroup analysis of the 10 most common toxic events.

**Fig. 4:**
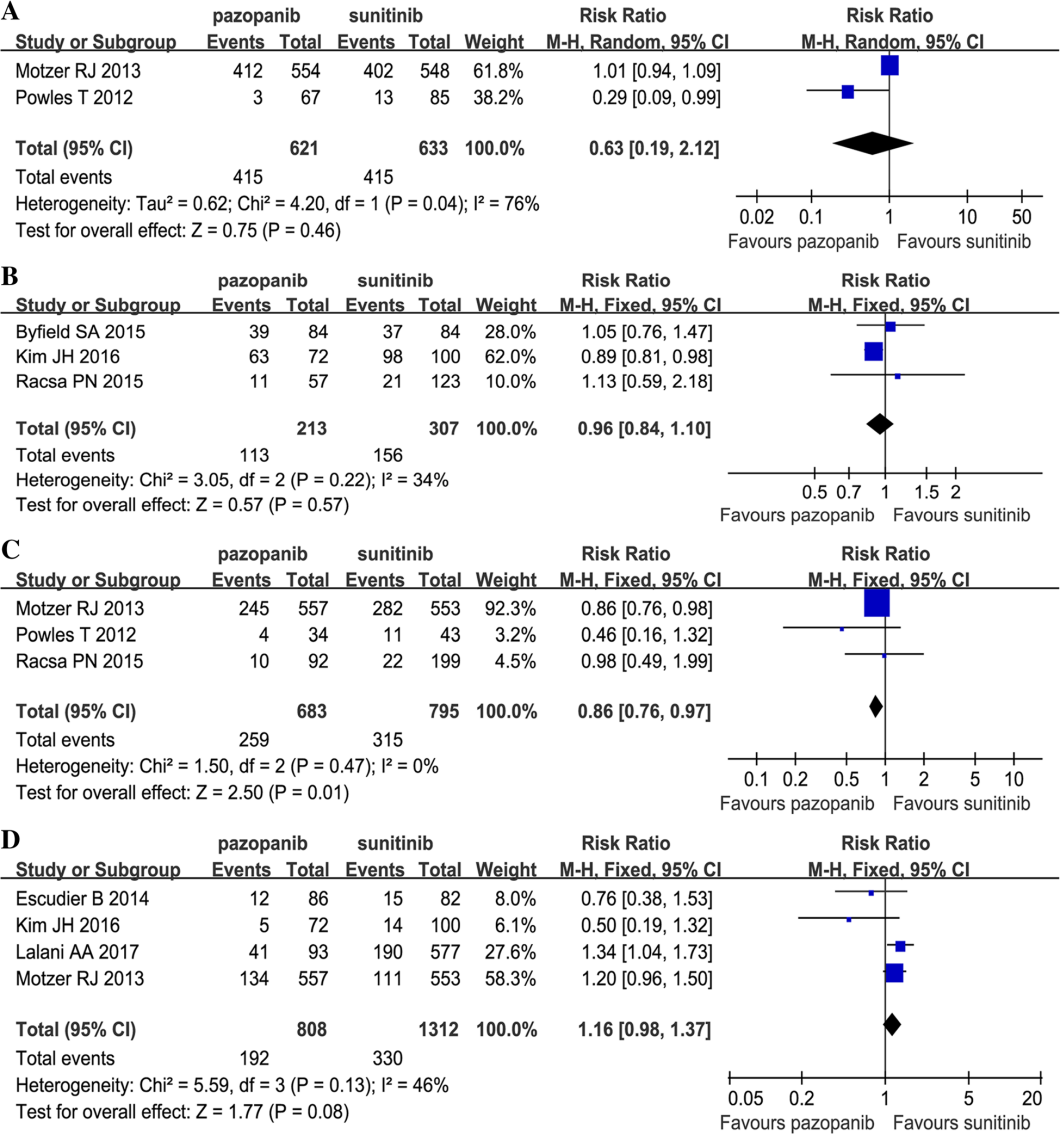
Forest plots of RR of grade 3–4 AEs (**a**), drug discontinuations (**b**), drug reductions (**c**) and drug discontinuations due to the serious AEs (**d**) associated with pazopanib versus sunitinib

Two articles compared grade 3–4 AEs (heterogeneity: *P =* 0.04, *I*^2^ = 76%). There was no significant difference between the two groups (RR = 0.63, 95% CI: 0.19–2.12, *P =* 0.46; Fig. 4A).

Some patients experienced drug discontinuation or reductions. Three studies compared drug discontinuations; there was no significant difference between the two groups (RR = 0.96, 95% CI: 0.84–1.10, *P =* 0.57; Fig. 4B). Three studies compared drug reductions; the sunitinib group had more drug reductions (RR = 0.86, 95% CI: 0.76–0.97, *P =* 0.01; Fig. 4C). Four studies compared drug discontinuations due to serious AEs; no significant difference was found (RR = 1.16, 95% CI: 0.98–1.37, *P =* 0.08; Fig. 4D).

In the subgroup analysis of the 10 most common AEs (diarrhea, fatigue, hypertension, nausea/vomiting, leukopenia, thrombocytopenia, neutropenia, increased creatinine, increased aspartate aminotransferase [AST], and increased alanine aminotransferase [ALT]), the outcomes of these grade 3–4 AEs indicated that there were no significant differences in hypertension, nausea/vomiting, and increased AST between the two groups. For all grade AEs, sunitinib had higher incidences of fatigue (RR = 0.87, 95% CI: 0.79–0.96, *P =* 0.006), leukopenia (RR = 0.55, 95% CI: 0.50–0.61, *P* < 0.00001), thrombocytopenia (RR = 0.54, 95% CI: 0.48–0.60, *P* < 0.00001), neutropenia (RR = 0.54, 95% CI: 0.48–0.61, *P* < 0.00001), and increased creatinine (RR = 0.68, 95% CI: 0.59–0.79, *P* < 0.00001); pazopanib induced significantly higher rates of diarrhea (RR = 1.11, 95% CI: 1.01–1.23, *P* = 0.03) and increased ALT (RR = 1.35, 95% CI: 1.20–1.51, *P* <  0.00001, Table 2). The outcomes of these grade 3–4 AEs demonstrated no significant differences were found for diarrhea, hypertension, nausea/vomiting, leukopenia, and increased creatinine between pazopanib and sunitinib. For grade 3–4 AEs, sunitinib had more fatigue (RR = 0.59, 95% CI: 0.44–0.80, *P=* 0.0006), thrombocytopenia (RR = 0.16, 95% CI: 0.10–0.25, P <  0.00001), and neutropenia (RR = 0.23, 95% CI: 0.15–0.34, P <  0.00001), but pazopanib had significantly higher incidences of increased AST (RR = 4.46, 95% CI: 2.62–7.58, P <  0.00001) and increased ALT (RR = 4.34, 95% CI: 2.79–6.75, P <  0.00001; Table 3).

**Table 2:** Top 10 adverse effects (all grade) associated with pazopanib versus sunitinib

Adverse effects	The number of study	Pazopanib group (event/total)	Sunitinib group (event/total)	RR (95% CI)	*P* value	Heterogeneity	
						*I*^*2*^ (%)	*P* value
Diarrhea	3	378/714	413/1210	1.11 [1.01–1.23]	0.03	11	0.32
Fatigue	3	350/714	542/1190	0.87 [0.79–0.96]	0.006	0	0.64
Hypertension	4	761/1241	783/1747	1.04 [0.99–1.10]	0.15	54	0.09
Nausea/Vomiting	3	434/719	531/1225	0.95 [0.88–1.02]	0.15	80	0.006
Leukopenia	2	258/626	477/648	0.55 [0.50–0.61]	< 0.00001	0	0.90
Thrombocytopenia	3	259/719	529/1225	0.54 [0.48–0.60]	< 0.00001	0	0.47
Neutropenia	3	227/719	447/1225	0.54 [0.48–0.61]	< 0.00001	0	0.57
Increased creatinine	2	190/626	284/648	0.68 [0.59–0.79]	< 0.00001	0	0.35
Increased AST	2	359/626	358/648	1.02 [0.93–1.12]	0.67	0	0.96
Increased ALT	2	351/626	266/648	1.35 [1.20–1.51]	< 0.00001	11	0.29

Abbreviations: ALT: alanine aminotransferase, AST: aspartate aminotransferase

**Table 3:** Top 10 adverse effects (3–4 grade) associated with pazopanib versus sunitinib

Adverse effects	The number of study	Pazopanib group (event/total)	Sunitinib group (event/total)	RR (95% CI)	P value	Heterogeneity	
						*I*^*2*^ (%)	P value
Diarrhea	3	51/660	43/691	1.19 [0.81–1.74]	0.38	0	0.42
Fatigue	3	60/660	102/691	0.59 [0.44–0.80]	0.0006	0	0.49
Hypertension	2	83/626	93/648	0.43 [0.05–3.53]	0.43	78	0.03
Nausea/Vomiting	3	25/660	29/691	0.87 [0.52–1.46]	0.61	0	0.61
Leukopenia	2	6/626	39/648	0.26 [0.03–2.55]	0.25	84	0.01
Thrombocytopenia	2	20/626	131/648	0.16 [0.10–0.25]	< 0.00001	0	0.37
Neutropenia	2	28/626	127/648	0.23 [0.15–0.34]	< 0.00001	0	0.97
Increased creatinine	2	4/626	8/648	0.49 [0.15–1.63]	0.25	NA	NA
Increased AST	2	71/626	16/648	4.46 [2.62–7.58]	< 0.00001	0	0.69
Increased ALT	2	99/626	23/648	4.34 [2.79–6.75]	< 0.00001	0	0.40

Abbreviations: ALT: alanine aminotransferase, AST: aspartate aminotransferase, NA: not available

### PPPM

**Fig. 5:**
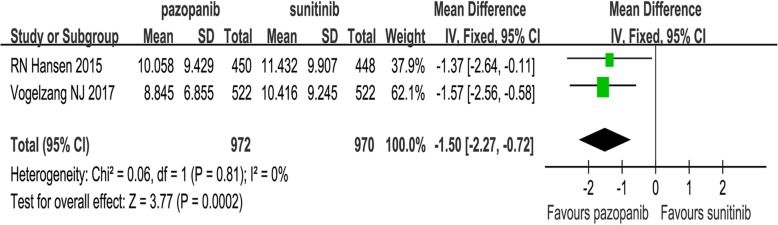
Forest plots of WMD of PPPM associated with pazopanib versus sunitinib

We assessed total costs between the pazopanib and sunitinib groups based on the PPPM. Two studies compared PPPM (heterogeneity: *P =* 0.81, *I*^2^ = 0%); the pazopanib group had significantly lower PPPM (WMD = − 1.50 thousand US dollars, 95% CI: − 2.27 to − 0.72, *P =* 0.0002; Fig. 5).

### Subgroup analysis

To determine if the anti-tumor effectiveness of pazopanib vs. sunitinib would be consistent across subgroups, the pooled results for PFS, OS, and ORR were calculated according to country, the number of patients in pazopanib group, risk classification, and study design (Table 4). Interestingly, the pooled results of included studies from the US found pazopanib had longer OS (HR = 0.86, 95% CI: 0.77–0.95, *P* = 0.004) and higher ORR (RR = 1.24, 95% CI: 1.03–1.51, *P* = 0.03), the pooled results of included studies from Korea also found pazopanib had improved OS (HR = 0.70, 95% CI: 0.49–0.99, *P* = 0.04), and the pooled results of RCT found pazopanib had better ORR (HR = 1.19, 95% CI: 1.00–1.43, *P* = 0.05) although the difference wasn’t significant.

**Table 4:** Subgroup analysis for progression-free survival, overall survival and objective response rate

Group	PFS				OS				ORR			
	No.of studies	HR (95% CI)	*P*	*I*^*2*^ (%)	No.of studies	HR (95% CI)	*P*	*I*^*2*^ (%)	No.of studies	RR (95% CI)	*P*	*I*^*2*^ (%)
Total	3	1.06 [0.98, 1.15]	0.13	0	8	0.92 [0.79–1.07]	0.29	61	8	1.03 [0.93, 1.13]	0.58	48
Nation												
USA	1	1.05 [0.90, 1.22]	0.53	NA	4	0.86 [0.77, 0.95]	0.004	28	2	1.24 [1.03, 1.51]	0.03	0
Canada	1	1.08 [0.98, 1.19]	0.12	NA	2	1.25 [0.78, 1.98]	0.35	75	1	0.91 [0.81, 1.04]	0.16	NA
Korea	1	0.91 [0.64, 1.30]	0.62	NA	1	0.70 [0.49, 0.99]	0.04	NA	1	1.57 [0.98, 2.52]	0.06	NA
Italy	NA	NA	NA	NA	1	0.94 [0.38, 2.32]	0.89	NA	2	1.28 [0.90, 1.82]	0.17	NA
UK	NA	NA	NA	NA	NA	NA	NA	NA	1	1.05 [0.35, 3.16]	0.93	NA
France	NA	NA	NA	NA	NA	NA	NA	NA	1	0.89 [0.48, 1.63]	0.70	NA
The number of pazopanib												
>100	2	1.07 [0.99, 1.16]	0.1	0	3	0.93 [0.81, 1.06]	0.26	60	2	1.05 [0.78, 1.42]	0.73	85
<100	1	0.91 [0.64, 1.30]	0.62	NA	5	0.95 [0.65, 1.38]	0.77	69	6	1.25 [0.96, 1.62]	0.09	0
classification a												
Poor risk	1	0.91 [0.64, 1.30]	0.62	NA	2	0.90 [0.56, 1.44]	0.66	78	1	1.57 [0.98, 2.52]	0.06	NA
Intermediate risk	NA	NA	NA	NA	2	1.36 [0.73, 2.52]	0.33	73	NA	NA	NA	NA
Mixed group	2	1.07 [0.99, 1.16]	0.1	0	7	0.95 [0.82, 1.11]	0.54	61	7	1.01 [0.91, 1.11]	0.85	43
Study design												
RS	2	1.07 [0.97, 1.17]	0.17	0	7	0.93 [0.77, 1.12]	0.44	67	5	1.17 [0.85, 1.61]	0.33	58
RCT	1	1.05 [0.90, 1.22]	0.53	NA	1	0.91 [0.76, 1.08]	0.29	NA	3	1.19 [1.00, 1.43]	0.05	0

Abbreviations: PFS: progression-free survival, OS: overall survival, ORR: objective response rate, HR, hazard ratio, RR: relative risk, RS: retrospective study, RCT: randomized controlled trial, NA: not available

**a** Patients were classified according to the International mRCC Database Consortium (IMDC) risk group

### Sensitivity analysis

PFS (Additional file 2: Figure S1A), OS (Additional file 2: Figure S1B), and DCR (Additional file 3: Figure S2B) were all robust: sensitivity analysis showed consistent results. However, the sensitivity analysis of ORR (Additional file 3: Figure S2A) showed that the estimate of the study Ruiz-Morales et al. [18] exceeded the 95% CI.

### Publication Bias

There was no proof of publication bias in PFS (Begg’s test, *p* = 0.296, Egger’s test, *P* = 0.058; Additional file 4: Figure S3A), OS (Begg’s test, *P* = 0.902; Egger’s test, *P* = 0.951; Additional file 4: Figure S3B), ORR (Begg’s test, *P* = 0.536; Egger’s test, *P* = 0.904; Additional file 5: Figure S4A), and DCR (Begg’s test, *P* = 0.806; Egger’s test, *P* = 0.479; Additional file 5: Figure S4B).

## Discussion

This is the first meta-analysis of the anti-tumor effectiveness, toxicity, and PPPM between pazopanib and sunitinib for treating mRCC or aRCC. Our analysis of 14 medium- to high-quality studies showed the two TKIs had equivalent anti-tumor effectiveness (PFS, OS, ORR, DCR), but sunitinib was associated with more all-grade/grade 3–4 fatigue, thrombocytopenia, neutropenia and higher PPPM. Additionally, pazopanib had more serious liver toxicity. In subgroup analysis, the pooled outcomes of US studies suggested that pazopanib may have longer OS and higher ORR.

Anti-tumor effectiveness is the most predominant cornerstone to consider when comparing pazopanib and sunitinib. The pooled analysis indicated no significant differences for OS, PFS, ORR, and DCR between pazopanib and sunitinib. A phase III RCT indicated pazopanib had comparable anti-tumor efficacy compared with sunitinib [7]. Furthermore, a retrospective observational study on the experiences of two Turkish hospitals demonstrated that pazopanib and sunitinib were similarly effective for treating mRCC [24]. Similarly, an article with 10-year results from a single-center study found no intergroup differences for treatment effectiveness [25]. Notably, subgroup analysis showed that the US studies had longer OS and higher ORR (Table 4), which suggested that pazopanib might have better anti-tumor effectiveness than sunitinib among American patients with mRCC or aRCC. The pooled results of Korean studies (95% CI: 0.49–0.99, *P* = 0.04) also indicated that pazopanib may prolong OS, but the limited number of studies (i.e., one) might weaken the certainty of this result. Additionally, the pooled results of RCT revealed pazopanib may better ORR although the difference wasn’t significant. Nevertheless, these conclusions of sub-analysis need be accepted carefully and require further large-sample, well-designed RCTs for confirmation.

The effect of drug toxicity is a significant factor when choosing pazopanib or sunitinib. Here, we observed high rates of drug reduction, diarrhea, fatigue, and hematologic toxicity (leukopenia, thrombocytopenia, neutropenia, increased creatinine) in the sunitinib group. A probable reason might be the inappropriate use of contemporary dose and schedule alterations when using sunitinib. In fact, in a phase-II RCT, Lee et al. found that therapy with 50 mg sunitinib daily using a 2/1 dosing schedule (2 weeks on; 1 week off) offered fewer AEs among mRCC patients than the standard 4/2 schedule (4 weeks on; 2 weeks of) (NCT00570882) after 30 months follow-up [26]. Simalarily, a retrospective analysis showed that therapy with 50 mg sunitinib daily using a 2/1 dosing schedule was associated with better tolerability and less toxicity among patients with mRCC as compared with the standard 4/2 schedule [27]. Although their survival time might not have been decreased, sunitinib-treated patients had slightly fewer clinical benefits based on quality-adjusted time without symptoms or toxicity (Q-TWiST) scores, which were used for evaluating the survival quality of patients, especially when the quality of life associated with tumor progression was higher than the quality of life related to AEs [28, 29]. In fact, this difference may be of vital importance for patients, as treatment was administered continuously over months [8]. However, liver toxicity (grade 3–4) was more frequent in the pazopanib group [7]. It was necessary to use hepatic protection drugs and monitor liver function periodically in patients using pazopanib, which may have helped prevent more severe hepatotoxic AEs. Similarly, periodic hematological examination and other relevant tests for sunitinib-treated patients were also significant. Moreover, in PISCES, a randomized, double-blind, crossover clinical trial comparing the influences of the two drugs’ toxicity and tolerability on the preferences of patients diagnosed with mRCC, 70% of patients and 61% of physicians preferred pazopanib over sunitinib [21]. In the COMPARZ trial, pazopanib-treated patients had superior health-related quality-of-life scores for both primary end points (fatigue and treatment AEs) during the first 6 months of therapy compared with sunitinib-treated patients [30]. In fact, physicians needed to be exceedingly cautious in administering pazopanib to patients with poor liver function.

The effect of total costs is also an indispensable factor when choosing between the two TKIs. Our results also proved that pazopanib had a significantly lower PPPM than sunitinib (Fig. 5). Delea et al. suggested that pazopanib was more cost-effective compared with sunitinib when used as first-line treatment among American patients with mRCC [31]. Other studies have reported similar results [18, 32–35]. Although the two TKIs did not have greatly different costs, the lower costs of pazopanib therapy might help relieve the financial burden on patients and their family, extend treatment time, and even relieve the psychological pressure on patients facing with so high expenditure, especially patients from impoverished families and from the developing countries.

The sensitivity analysis was performed to analyze the moderate-significant heterogeneity (I2 > 50%) of OS, ORR and DCR. We assumed that Lalani AA et al. [9] in OS, Ruiz-Morales et al. [18] in ORR, and Kim JH et al. [13] in DCR might be the drivers of the moderate-significant heterogeneity. In the study Lalani AA et al. [9], there were less patients with Karnofsky performance score < 80 in the sunitinib group than the pazopanib group (17% vs. 26%), and they would have longer survival. As a result, sunitinib-treated patients of the study Lalani AA et al. [9] had better OS. In addition, the sensitivity analysis of ORR showed that this result was not very robust, and the estimate of the study Ruiz-Morales et al. [18] exceeded the 95% CI. We found that patients in the sunitinib group were younger than pazopanib-treated patients (median age: 62 vs. 65, *P* <  0.0001) in the study of Ruiz-Morales et al. [18], which might cause better ORR. In a retrospective study, Hutson et al. reported more treatment-emergent AEs were recorded in older patients using sunitinib [36]. Older patients might have more drug discontinuation or reductions, which inevitably influenced response rate of targeted drugs. Therefore, sunitinib-treated patients of the study Ruiz-Morales et al. [18] had better ORR. Additionally, we found that there were all poor-risk patients with mRCC in the study Kim JH et al. [13], which might give rise to significantly poor DCR of sunitinib-treated patients.

Several limitations should be considered when considering our results. First, the limited number of RCTs (only three) weakened the quality of these outcomes and the inclusion of retrospective studies would exert a certain impact on the reliability of these outcomes. Admittedly, it might increase the heterogeneity when we analyzed PFS and OS if we combined different types of studies. But we had performed the subgroup analysis of PFS and OS about study design in our meta-analysis, and no difference was found in this subgroup analysis between the two groups in terms of PFS and OS. Therefore, the bias caused by the combination of the two different types of studies might be relatively small. Second, there was moderate-significant heterogeneity for some comparisons (OS, ORR and DCR), which weakened the reliability of these outcomes. Third, the limited number of studies on PFS, PPPM, and the subgroup of OS in Korea might have resulted in relatively unreliable estimates. Fourth, the included articles were limited to literature sources published in English, so it might lead to the language bias. However, as a comprehensive and universal language, English studies have more reliable information, so we can guarantee the quality of all included studies if the only English studies were included. Fifth, we could not control completely for confounding factors (pre-treatment, pathological type), which were unavailable for some studies but which could influence final results. Sixth, some specific subsequent costs were part of PPPM, but they didn’t have direct association with the anti-tumor effectiveness. Accordingly, we suggested that total healthcare costs should be elaborated if possible in future drug evaluation studies.

Our meta-analysis showed that pazopanib had similar anti-tumor effectiveness for mRCC or aRCC as compared with sunitinib. But pazopanib may be more suitable to poor patients due to lower PPPM. Great care should be taken when using pazopanib to treat patients with abnormal liver function. In addition, pazopanib might have more benifits (better OS, higher ORR) among American patients with mRCC or aRCC. Nevertheless, the inherent limitations of this meta-analysis mean that additional large-scale, high-quality studies are required to better determine the role of the two targeted drugs under complicated clinical circumstances.

## Additional files

Additional file 1: **Table S1.** PRISMA 2009 Checklist. (DOC 65 kb)

Additional file 2: **Figure S1.** Sensitivity analysis of PFS (A) and OS (B) (TIF 22670 kb)

Additional file 3: **Figure S2.** Sensitivity analysis of ORR (A) and DCR (B) (TIF 1268 kb)

Additional file 4: **Figure S3.** Begg’s and Egger’s tests for comparisons of HR of PFS (A) and OS (B) associated with pazopanib versus sunitinib. (TIF 8671 kb)

Additional file 5: **Figure S4.** Begg’s and Egger’s tests for comparisons of ORR (A) and DCR (B) associated with pazopanib versus sunitinib. (TIF 1210 kb)

## Abbreviations

AEs: Adverse effects
ALT: Alanine aminotransferase
aRCC: Advanced renal cell carcinoma
AST: Aspartate aminotransferase
CIs: Confidence intervals
DCR: Disease control rate
HRs: Hazard ratios
mRCC: Metastatic renal cell carcinoma
ORR: Objective response rate
OS: Overall survival
PFS: Progression-free survival
PPPM: Per-patient-per-month costs
RCT: Randomized controlled trial
RRs: Risk ratios
TKI: Tyrosine kinase inhibitor
WMD: Weighted mean difference
