# Supplementary figures and images for: Pazopanib has equivalent anti-tumor effectiveness and lower Total costs than Sunitinib for treating metastatic or advanced renal cell carcinoma: a meta-analysis
Source: BMC Cancer. 2019 May 23;19:489. doi: 10.1186/s12885-019-5704-3 (PMC6533682; doi:10.1186/s12885-019-5704-3)

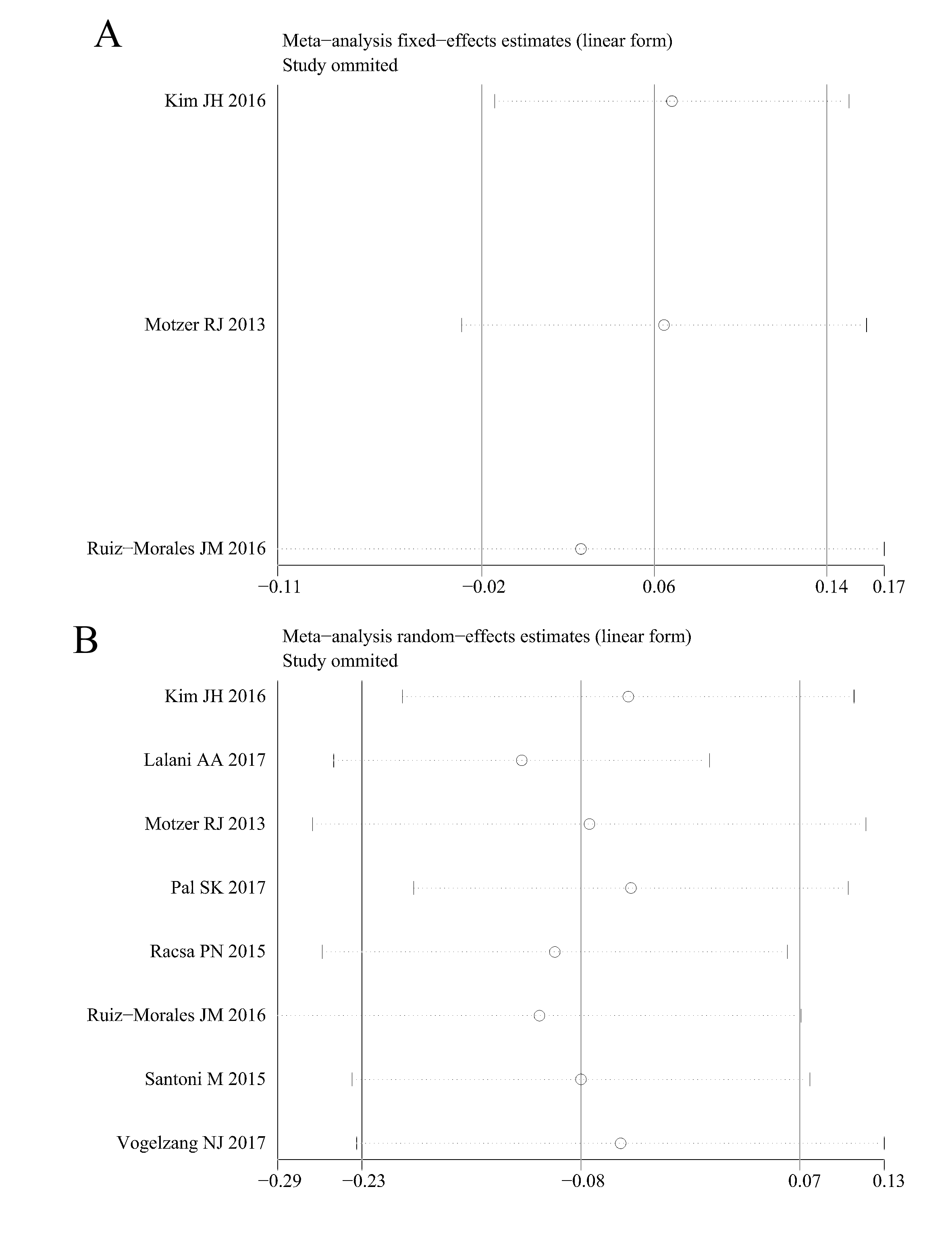

Supplement: Supplementary file 2 — Figure S1. Sensitivity analysis of PFS (A) and OS (B) (TIF 22670 kb) [file 12885_2019_5704_MOESM2_ESM.tif]

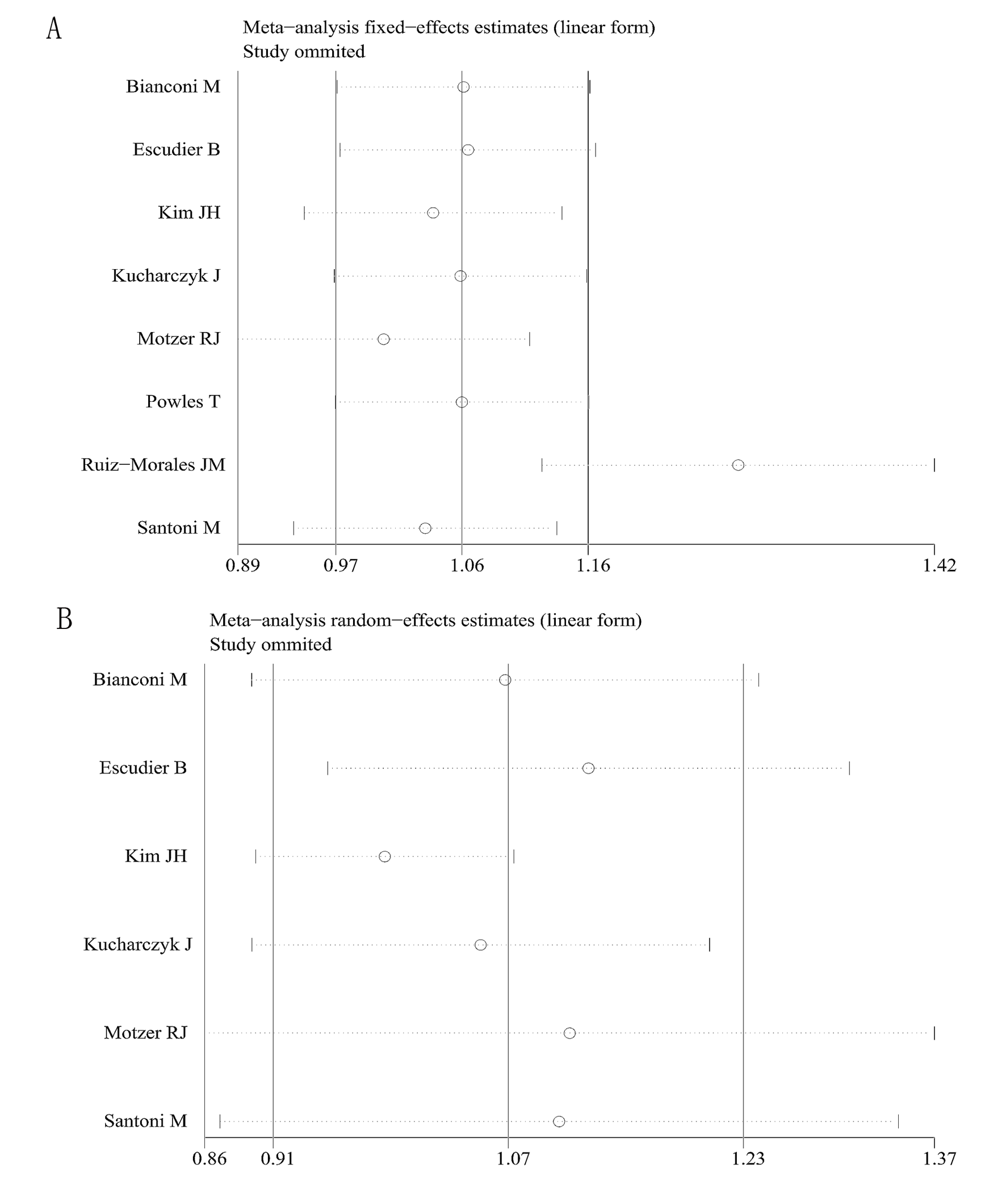

Supplement: Supplementary file 3 — Figure S2. Sensitivity analysis of ORR (A) and DCR (B) (TIF 1268 kb) [file 12885_2019_5704_MOESM3_ESM.tif]

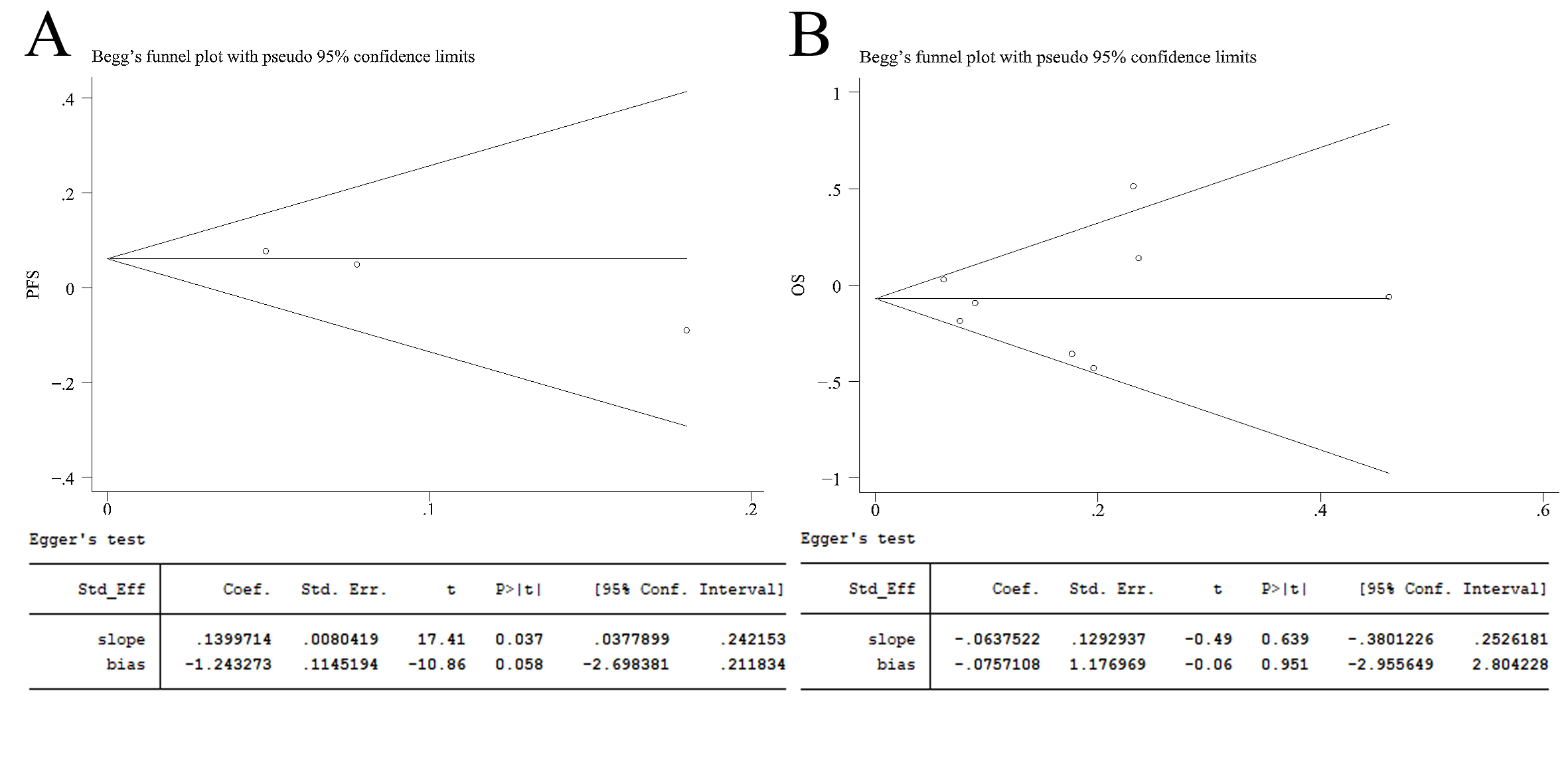

Supplement: Supplementary file 4 — Figure S3. Begg’s and Egger’s tests for comparisons of HR of PFS (A) and OS (B) associated with pazopanib versus sunitinib. (TIF 8671 kb) [file 12885_2019_5704_MOESM4_ESM.tif]

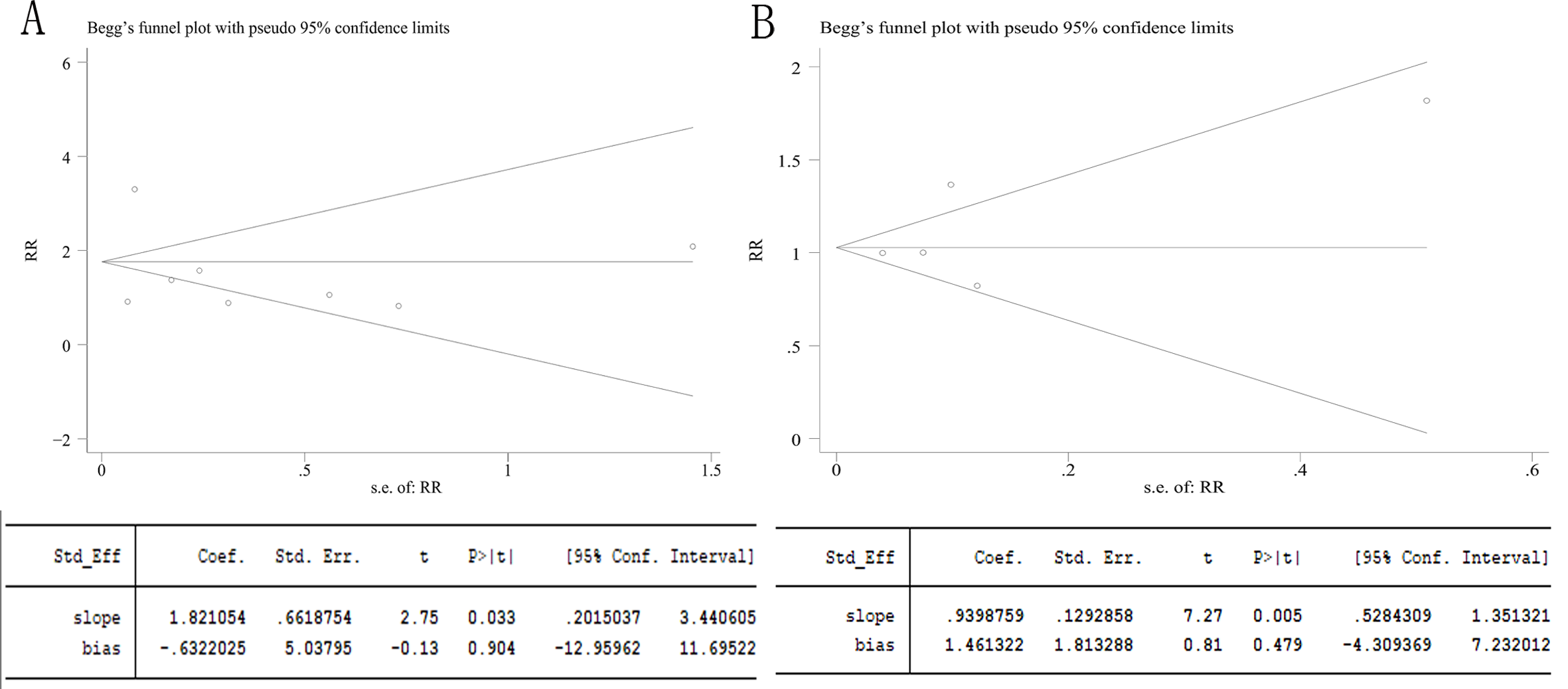

Supplement: Supplementary file 5 — Figure S4. Begg’s and Egger’s tests for comparisons of ORR (A) and DCR (B) associated with pazopanib versus sunitinib. (TIF 1210 kb) [file 12885_2019_5704_MOESM5_ESM.tif]
